# DGCR8-Mediated Production of Canonical Micrornas Is Critical for Regulatory T Cell Function and Stability

**DOI:** 10.1371/journal.pone.0066282

**Published:** 2013-05-31

**Authors:** Lukas T. Jeker, Xuyu Zhou, Robert Blelloch, Jeffrey A. Bluestone

**Affiliations:** 1 Diabetes Center and the Department of Medicine, University of California San Francisco, San Francisco, California, United States of America; 2 Department of Pathology, University of California San Francisco, San Francisco, California, United States of America; 3 The Eli and Edythe Broad Center of Regeneration Medicine and Stem Cell Research, Center for Reproductive Sciences, University of California San Francisco, San Francisco, California, United States of America; 4 Department of Urology, University of California San Francisco, San Francisco, California, United States of America,; University of Southern California, United States of America

## Abstract

Regulatory T cells (Treg) are integral for immune homeostasis. Here we demonstrate that canonical microRNAs (miRNAs) are required for Treg function because mice with DGCR8-deficient Treg cells spontaneously develop a scurfy-like disease. Using genetic lineage marking we show that absence of miRNAs leads to reduced FoxP3 expression in Treg cells in vivo. In vitro culture of purified DGCR8-deficient Treg leads to a loss of FoxP3 expression. We conclude that canonical miRNAs are essential to maintain stable FoxP3 expression and Treg function. Thus, signals interfering with miRNA homeostasis might contribute to autoimmune diseases.

## Introduction

Immune regulation depends largely on regulatory T cells (Treg). These CD4^+^ helper T cells express the forkhead domain transcription factor FoxP3. Mutations interfering with FoxP3 function lead to a rapidly lethal inflammatory syndrome called Immune dysregulation, Polyendocrinopathy, Enteropathy X-linked (IPEX) in humans and scurfy in mice 1]. Treg suppress proinflammatory cells using multiple mechanisms 2]. Treg do not produce Interleukin-2 (IL-2) or other lineage-specific proinflammatory cytokines such as Interferon-γ (IFNγ), Interleukin-17 (IL-17) or IL-4. However, in the past few years, it has become commonly accepted that T cell lineage commitment is not as rigid as previously thought 3]. Multiple studies have challenged that Treg are stable under all circumstances 4,5,6,7,8,9] and the molecular program underlying Treg lineage commitment and maintenance of their stability is under intense investigation 10,11,12]. We have recently demonstrated that some Treg can lose FoxP3 expression and turn into pathogenic cells 5]. Thus, understanding Treg stability is highly relevant for clinical trials involving therapeutic adoptive transfer of various kinds of Treg 13,14].

Cellular differentiation and cell lineage stabilization can be reinforced post-transcriptionally through miRNAs 15,16,17]. Previous studies have shown that Treg-specific Dicer ablation results in a spontaneous, aggressive multiorgan inflammatory syndrome resembling scurfy disease 18,19,20]. However, Dicer ablation not only interrupts the generation of canonical miRNAs but also interferes with the generation of other small RNAs like endogenous siRNAs, endogenous shRNAs, mirtrons and Alu RNA 21], which can be functional in some mammalian cells 22,23,24]. DGCR8, a RNA-binding protein required for the specific processing of canonical miRNAs 21,25], is more specific for canonical miRNAs than Dicer 21]. Therefore, we used Treg-specific *Dgcr8* ablation to investigate the specific role of canonical miRNAs in Treg function and stability.

## Results

### Efficient and specific deletion of *Dgcr8* and miR-150

**Figure 1:**
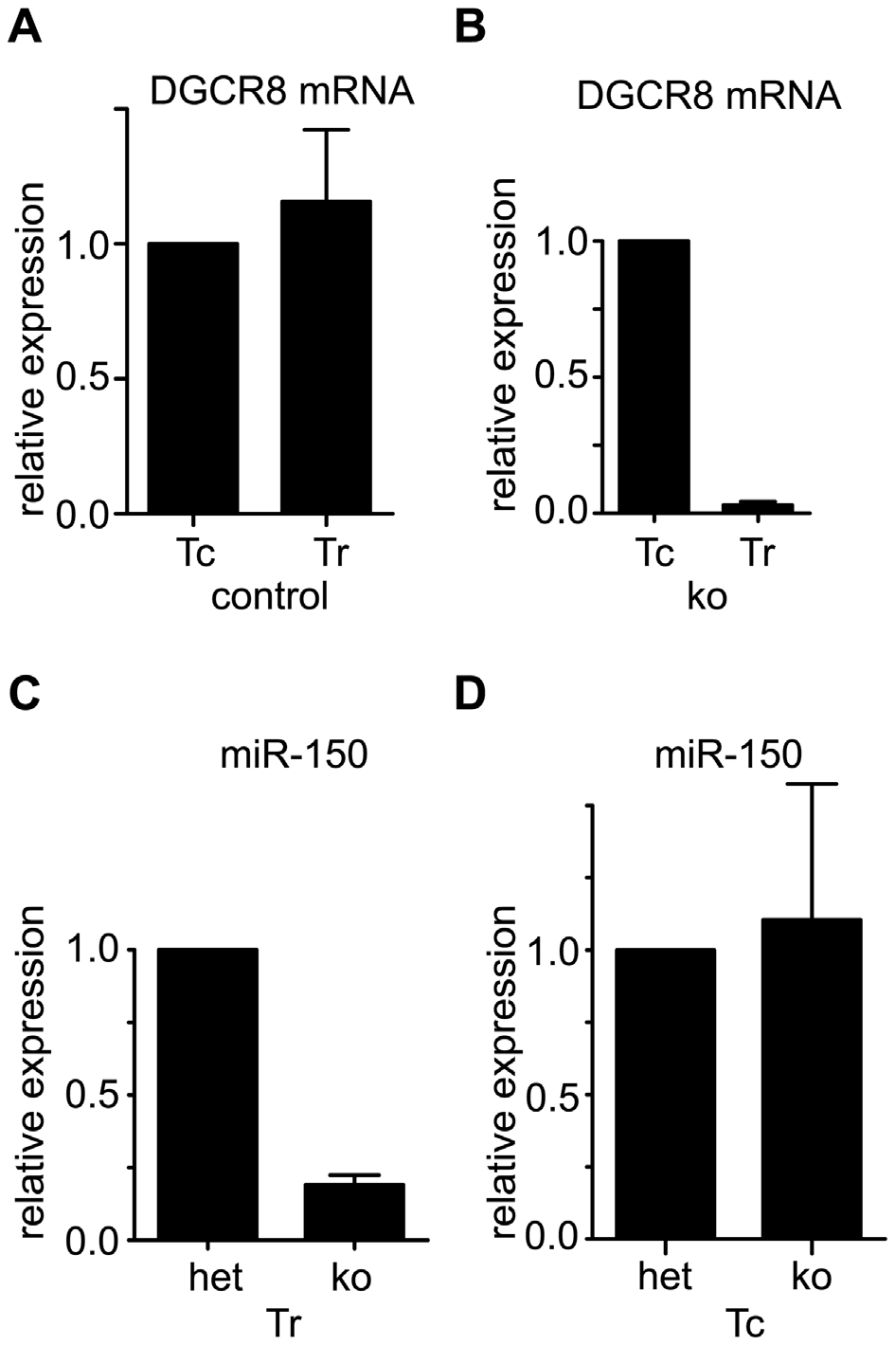
Efficient and Treg-specific deletion of *Dgcr8* and miR-150. Conventional CD4^+^GFP^−^ (Tc) and CD4^+^GFP^+^ regulatory (Tr) T cells were FACS sorted from FoxP3-GFP reporter mice (a) and FoxP3-GFP-hCre:DGCR8^wt/lox^ (het) or FoxP3-GFP-hCre:DGCR8^lox/lox^ (KO) mice (b-d). qPCR analysis of Dgcr8 mRNA in Tc and Tr (a,b) and miR-150 (c,d). Pooled data from 3 independent experiments with 3 mice total (a,b) and representative data from 2 independent experiments with 2 mice total (c,d).

Purified conventional (Tconv) and regulatory CD4^+^ T cells express comparable levels of *Dgcr8* mRNA (**Fig. 1a**). To interrogate the functional role of canonical miRNAs in Treg in vivo we ablated *Dgcr8* specifically in Treg using a previously described FoxP3-GFP-hCre BAC transgene 18]. *Dgcr8* was specifically and efficiently ablated in Treg (**Fig. 1b**). The microRNA miR-150 as a representative canonical miRNA was specifically ablated in Treg (**Fig. 1c**) but not conventional CD4^+^ T cells (**Fig. 1d**). The specificity and efficiency were comparable to *Dicer* ablation (data not shown and Ref 18]). Mice with Treg-specific *Dgcr8* heterozygosity expressed approximately 50% reduced *Dgcr8* levels in Treg but did not affect miRNA expression and were therefore used as controls in some of the experiments (data not shown).

### Absence of canonical miRNAs in Treg leads to a spontaneous scurfy-like lethal syndrome

**Figure 2:**
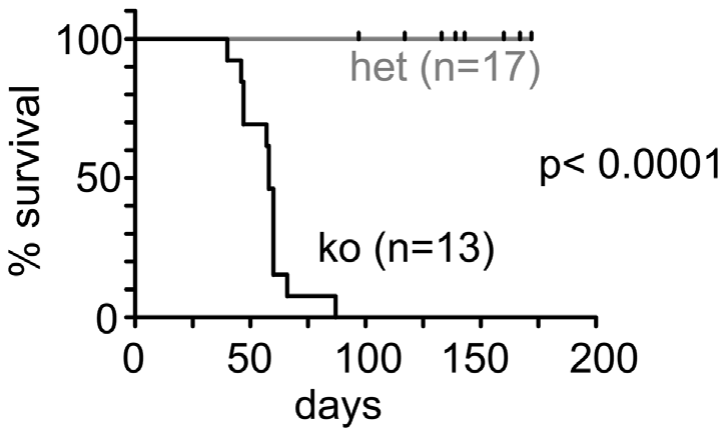
Early lethality in mice with a Treg-specific *Dgcr8* deficiency. Survival curve of pooled female and male FoxP3-GFP-hCre:DGCR8^wt/lox^ (het) or FoxP3-GFP-hCre:DGCR8^lox/lox^ (KO) mice. Mice found dead or required to be euthanized due to severe body condition as per institutional requirements were collectively flagged as “dead” for the analysis. P<0.0001 (Log-rank (Mantel-Cox) Test.

**Figure 3:**
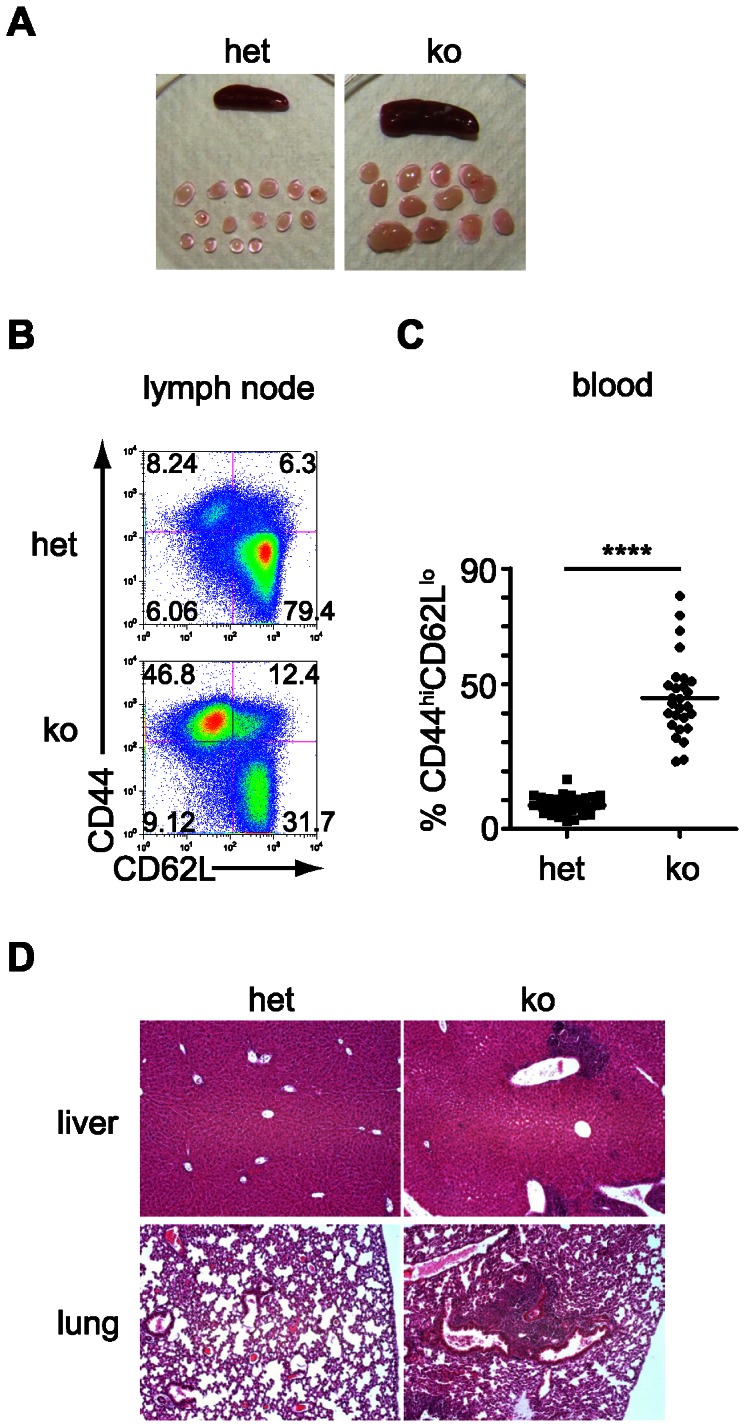
Mice with Treg lacking canonical miRNAs develop scurfy-like disease. Macroscopic (a), flow cytometric (b,c) and histologic (d) analysis of the disease spontaneously occurring in FoxP3-GFP-hCre:DGCR8^lox/lox^ (KO) mice. FoxP3-GFP-hCre:DGCR8^wt/lox^ (het) served as control mice. (a) Splenomegaly (top) and lymphadenopathy (bottom) in het and KO mice. (b) Lymph nodes (LN) were harvested from ≥3 week old het and KO mice. Single cell suspensions stained with anti-CD44 and anti-CD62L to determine activation of CD4^+^ Tconv (gated on CD4^+^GFP^−^) as an indirect readout of Treg function were analyzed by flow cytometry (FACS). The increased frequency of CD44^hi^CD62L^lo^ cells among CD4^+^GFP^−^ lymphocytes represents spontaneous activation of Tconv in lymph node cells. Representative FACS plots from >10 independent experiments. (c) Quantification of frequency of activated CD44^hi^CD62L^lo^ among CD4^+^GFP^−^ Tconv in peripheral blood of 3–4 week old mice. n = 35 (het), n = 27 (KO). p<0.0001 (Two-tailed Mann-Whitney Test). (d) Representative paraffin-embedded sections of liver and lung tissues stained with Hematoxylin & Eosin of het and KO mice. Pictures were taken with 100×optical magnification. 4/4 KO livers and 4/4 KO lungs had infiltrates, 0/4 het livers or lungs were infiltrated.

Mice lacking canonical miRNAs in Treg were small, runted, failed to thrive and died prematurely. Mortality started as early as 3 weeks after birth and reached 100% after 3 months (**Fig. 2**). The mice spontaneously developed lymphadenopathy and splenomegaly (**Fig. 3a**). Conventional CD4^+^GFP^−^ T cells (Tconv) displayed an activated phenotype (CD44^hi^CD62L^lo^) in lymph nodes and blood which was observed as early as at 3 weeks after birth (**Fig. 3b,c**), indicating that *Dgcr8*-deficient Treg were unable to control immune homeostasis. This resulted in infiltration of multiple organs including lungs and livers (**Fig. 3d**) and dramatic increase in the number of IFN-γ producing CD4^+^ and CD8^+^ T effector cells (data not shown).

### *Dgcr8* is required for Treg stability

**Figure 4:**
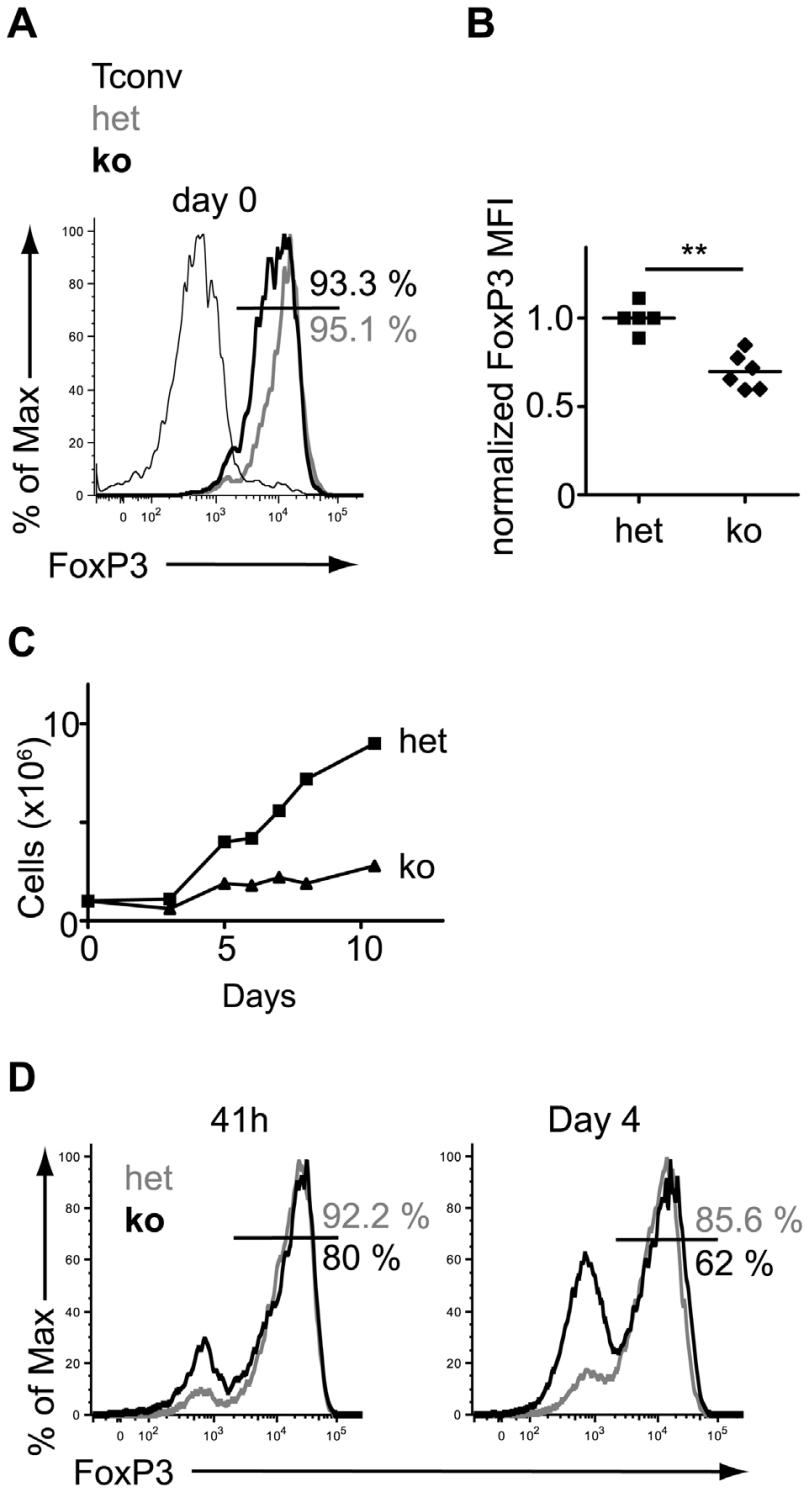
Canonical miRNAs stabilize FoxP3 expression. Flow cytometric analysis of FoxP3 stability in lymphocytes isolated from FoxP3-GFP-hCre:DGCR8^wt/lox^ (het) and FoxP3-GFP-hCre:DGCR8^lox/lox^ (KO) mice. (a) Representative FACS histograms of FoxP3 intracellular staining of CD4^+^GFP^−^ Tconv and CD4^+^GFP^+^ Treg with heterozygous (het) or homozygous deletion of Dgcr8 (KO) isolated from lymph nodes (LN). (b) Quantification of FoxP3 median fluorescence intensity (MFI) in het and KO Treg cells isolated from LN. The MFI was normalized due to inter-experimental variability of the relative FoxP3 MFI. For normalization the MFI of the control Treg was set to 1 and the relative reduction of MFI was calculated for the ko. In experiments with more than one control the mean MFI of the het controls was set to 1. In each individual experiment the het control Treg displayed a higher FoxP3 MFI than ko Treg. Statistical analysis was performed on pooled normalized data from four independent experiments. p = 0.0075 (Two-tailed Mann-Whitney Test). (c) FACS sorted CD4^+^GFP^+^ Treg were cultured with beads coated with anti-CD3 and anti-CD28 antibodies and 2000 U/ml IL-2 and live cells were counted. (d) Treg were purified and cultured under expansion conditions as described for panel c. At 41 h and after 4 days samples were stained for intracellular FoxP3 as in panel a. The bar indicates the gate used to define the cutoff for FoxP3 staining. Numbers indicate the % of FoxP3 expressing cells. Representative growth curve (c) and representative FACS plots (d) from 2 independent experiments.

Loss of and/or unstable FoxP3 expression can lead to the generation of pathogenic exFoxP3 cells 5]. Using a lineage tracing system we have previously shown that some Treg lacking DICER lose FoxP3 expression in vivo 18]. To test if canonical miRNAs were required for FoxP3 expression in Treg that are actively transcribing FoxP3, as evidenced by GFP reporter expression, we purified CD4^+^GFP^+^ cells from het and KO mice and subsequently stained for intracellular FoxP3 protein. The median FoxP3 fluorescence intensity among FoxP3 expressing cells was compromised in *Dgcr8*-deficient Treg as compared to miRNA-sufficient Treg (**Fig. 4a,b**). Next, we investigated FoxP3 stability of miRNA-deficient Treg cultured in vitro. Purified CD4^+^GFP^+^ Treg from het and KO mice were activated and expanded in vitro with beads coated with anti-CD3 and anti-CD28 and high doses of IL-2. The expansion of *Dgcr8* KO Treg was severely impaired (**Fig. 4c**), at least in part due to increased cell death (data not shown). Importantly, *Dgcr8*-deficient Treg progressively lost FoxP3 under these conditions despite the presence of high doses of IL-2 (**Fig. 4d**). These results mimicked those observed in *Dicer*-deficient Treg, which were previously shown to lose FoxP3 and start to produce potentially pathogenic cytokines which might contribute to the autoimmunity 18]. Thus, canonical miRNA-deficiency was sufficient to cause Treg instability.

## Discussion

Eliminating key proteins of the miRNA biogenesis pathway can be used to study global miRNA function. Selective ablation of *Dicer* in Treg leads to a profound and lethal multi-organ autoimmune syndrome 18,19,20]. However, although DICER cleaves miRNA precursors, its activity is not entirely specific for canonical miRNAs 21]. The DROSHA - DGCR8 miRNA microprocessor complex is critical for the cleavage of primary miRNA transcripts and DGCR8 is thought to be specific for the processing of canonical miRNAs rather than other classes of nuclear (small) RNAs 21] although it remains controversial if and to what degree the microprocessor complex also cleaves other RNAs including snoRNAs 26,27]. Furthermore, the DROSHA - DGCR8 miRNA microprocessor complex recognizes and cleaves *Dgcr8* mRNA in an autoregulatory loop mostly specific for the microprocessor itself 27,28,29] and DGCR8 stabilizes DROSHA through protein-protein interaction 28]. Thus, *Dgcr8*-deletion is mostly specific for canonical miRNAs. Herein, we demonstrate that *Dgcr8* is essential for Treg function and lineage stability. Our results parallel the observation that DROSHA is essential for Treg function 19]. Our findings do not exclude that non canonical miRNAs like mirtrons or other small RNAs are functional in Treg but they suggest that ablation of the pool of canonical miRNAs is sufficient to cause Treg dysfunction which in turn leads to breakdown of immune homeostasis. In addition, freshly isolated *Dgcr8*-deficient Tregs expressed slightly reduced FoxP3 levels and were unable to maintain FoxP3 expression when cultured in vitro. Furthermore, the frequency of *Dgcr8*-deficient GFP expressing Treg was reduced in peripheral blood compared to control mice (data not shown). However, the relative reduction was more pronounced in peripheral blood than lymph nodes. Moreover, initial studies suggest a discrepancy between GFP and FoxP3 protein expression. This could indicate that FoxP3 transcription (read out as GFP) is terminated prematurely in the absence of miRNAs. However, further investigation is required to address this interesting observation. Together, these results confirm and extend our findings that Dicer-deficient Treg lose FoxP3 expression as revealed with a genetic lineage tracing system 18]. Importantly, the T cell receptor repertoire of Treg is skewed towards self-reactivity 30,31]. Hence, loss of FoxP3 could result in self-reactive pathogenic effector T cells. Indeed, miRNA-sufficient exFoxP3 cells can be pathogenic upon adoptive transfer 5]. However, it is unclear whether miRNA-deficient exFoxP3 cells would be pathogenic. Although miRNAs regulate expression of about 50% of all genes 32] and are important for proliferation, survival and differentiation of most cell types, cells devoid of miRNAs can still be functional. As an example, cancer cells can be functional despite absence of miRNAs 33]. Likewise, CD4-cre transgene mediated ablation of *Dicer* or *Drosha* in all CD4^+^ and CD8^+^ T cells results in a spontaneous multiorgan inflammatory syndrome, likely due to impaired Treg function 19,34]. Importantly, *Dicer*- and *Dgcr8*-deficient Tconv are prone to Th1 responses with a strongly increased relative number of Tconv secreting IFN-γ compared to control cells 35,36]. However, mice with a miRNA deficiency in both Treg and Tconv cells do not develop full-blown FoxP3-deficiency disease as seen with Treg-specific *Dicer*, *Drosha* or *Dgcr8* ablation: disease starts much later and only affects some mice. This indicates that both Treg and Tconv cells are impaired functionally. Therefore, disease results from the net balance of miRNA-deficient effector T cells and miRNA-deficient Treg.

The rapid, fatal disease observed in mice with miRNA-deficient Treg likely reflects a complex collection of defects resulting from multiple missing individual miRNAs. miR-155 might stabilize Treg by facilitating IL-2 signaling 37] and miR-10a might stabilize the Treg lineage 38,39] but genetic deletion of either miRNA does not lead to substantial loss of FoxP3 indicating that other miRNAs or a combination of miRNAs must be stabilizing FoxP3. In addition, miRNAs are likely contributing to the characteristic absence of inflammatory cytokine production in Treg because *Dicer*-deficient Treg produce IFN-γ 18]. Since miR-29ab is a potent repressor of IFN-γ production in T cells 36,40] it might be involved in IFN-γ repression in Treg. Indeed, Treg express high levels of miR-29ab 41]. Since miR-29ab is induced by IFN-γ functioning in a negative feedback loop 42] it might be particularly important to prevent Treg from producing IFN-γ in an inflammatory setting. It will be interesting to test miR-29ab and other miRNA expression in human Treg expressing IFN-γ 43]. Finally, the availability of an increasing number of mouse models with genetic miRNA deficiencies will be important to study the complex miRNA functions in immune regulation in vivo 44,45].

In summary, canonical miRNAs are essential for normal FoxP3 expression and suppressive Treg function. Deciphering the function of individual miRNAs will be essential to define novel therapeutic targets. Furthermore, studying the function of individual miRNAs in Treg combined with a better understanding of the genetic networks regulated by those miRNAs holds the promise to yield new insight into Treg biology.

## Materials and Methods

### RNA isolation and RT-qPCR

Total RNA was isolated from cells lysed in 500 µl Trizol (Invitrogen) according to the manufacturer's recommendations. Glycogen (New England Biolabs) was used as a carrier. miRNA RT qPCR was done as described 39,46]. For mRNA analysis cDNA was generated using the High capacity cDNA reverse transcription kit according to the manufacturer's recommendations (Applied Biosystems). For detection of *Dgcr8* mRNA TaqMan Gene expression assay mus musculus *Dgcr8*, (Mm01146846_g1) was used (Applied Biosystems).

### Mice

Mice harboring a loxP flanked conditional DGCR8 allele (MGI: 3819698), Foxp3-GFP-hCre (MGI: 4430213), and FoxP3-GFP (MGI: 3574964) mice have been described 18,47,48]. Mice were housed and bred under specific pathogen-free conditions at the University of California, San Francisco (UCSF) Animal Barrier Facility. Animal experiments were approved by the Institutional Animal Care and Use Committee (IACUC) of UCSF (approval numbers AN083988-01 and AN082188-02).

### Antibodies, Flow cytometry and FACS sorting

Flow cytometry and fluorescence activated cell sorting was performed as described 5,18]. Labeled anti-CD4 (RM4-5), anti-CD8 (Ly-2), anti-CD44 (pgp-1), anti-CD62L (MEL14) and anti-Foxp3 (FJK-16s) antibodies were from eBioscience, Biolegend or BD Biosciences. FoxP3 staining was performed using a commercially available FoxP3 staining kit (eBioscience). Stained cells were analyzed with a FACS Calibur and CELLQuest software or on an LSR II and FACSDiva (BD Biosciences) for acquisition. Subsequent analysis was performed using FlowJo (TreeStar). T cells were sorted with a MoFlo high-speed cell sorter (DakoCytomation)

### Treg expansion conditions:

Treg were sorted on a MoFlo (Dako Cytomation) as CD4^+^CD8^−^GFP/YFP^+^ lymphocytes and expanded using Dynabeads coated with anti-CD3 and anti-CD28 (Invitrogen) in the presence of 2000U human IL-2/ml as described 49]. Cell to bead ratio was 1∶1. Cell culture medium was DMEM containing 10% (vol/vol) heat-inactivated fetal bovine serum (Biosource International), nonessential amino acids, 0.5 mM sodium pyruvate, 5 mM HEPES, pH 7.4, 1 mM GlutaMAX I (all from Invitrogen) and 55 µM ß-mercaptoethanol (Sigma-Aldrich)).

### Histology

Lungs and livers were fixed in 10% formalin overnight. After washing in PBS tissues were dehydrated in 40% Ethanol for 5′, then kept in 70% Ethanol until paraffin embedding. Paraffin embedding was done using a Leica tissue processor. Tissues were then stained with hematoxylin and eosin.

### Statistics

For statistical analysis Prism 5.0 (GraphPad Software) was used. * denotes p≤0.05, **denotes p≤0.01, ***denotes p≤0.001, **** denotes p≤0.0001. Differences between groups were considered statistically significant if the null hypothesis was rejected by a p value of ≤5%.
